# Using Local Anesthesia for Burr Hole Surgery of Chronic Subdural Hematoma Reduces Postoperative Complications, Length of Stay, and Hospitalization Cost: A Retrospective Cohort Study From a Single Center

**DOI:** 10.3389/fsurg.2022.783885

**Published:** 2022-04-01

**Authors:** Zerui Zhuang, Zelin Chen, Hui Chen, Bin Chen, Jianzhi Zhou, Anmin Liu, Jianming Luo

**Affiliations:** ^1^Department of Neurosurgery, Sun Yat-Sen Memorial Hospital, Sun Yat-Sen University, Guangzhou, China; ^2^Department of Neurosurgery, Shantou Central Hospital, Shantou, China; ^3^Department of Neurosurgery, Second Affiliated Hospital, Shantou University Medical College, Shantou, China

**Keywords:** chronic subdural hematoma (CSDH), local anesthesia (LA), general anesthesia (GA), burr hole surgery, elderly patients

## Abstract

**Purpose:**

The purpose of the current study was to compare the effects of local anesthesia (LA) and general anesthesia (GA) on the surgical process and postoperative recovery of patients with unilateral chronic subdural hematoma (CSDH).

**Patients and Methods:**

A retrospective cohort study was conducted on patients with unilateral CSDH who underwent burr hole surgery between the years 2013 and 2018. Patients who received local anesthesia were allocated to the LA group, and the patients who received general anesthesia were allocated to the GA group. The clinical data, postoperative complication, length of stay, and hospitalization cost of these two groups were compared and analyzed.

**Results:**

Data from 105 patients was collected for this study. Fifty one patients were assigned to the LA group and 54 to GA group. The duration of anesthesia and operation of the LA group was 37.71 (10.55) min; while for the GA group the duration was 56.04 (8.37) min (*p* < 0.001). The time from operation to discharge in GA group was greatly longer than that in LA group [(8.51 (1.49) days vs. 10.46 (2.34) days, respectively; *p* < 0.001]. Hospitalization cost for LA group was 2,721.54 (504.66) USD, which was significantly lesser than that for GA patients [3,314.82 (493.52) USD; *p* < 0.001]. The total number of complications in LA patients was less than that in GA patients [6 vs. 29 cases, respectively; *p* < 0.001]. The number of patients with residual hematoma in the LA group was <that in the GA group (*p* = 0.014).

**Conclusion:**

As compared to GA, LA might be a simpler, safer, and more effective method for burr hole surgery of CSDH to promote patients' recovery. However, further research is still required to confirm this conclusion.

## Introduction

Chronic subdural hematoma (CSDH) is a common neurosurgical disease, and the increase is shown as the population aging nowadays. Studies have predicted that CSDH will become the most common brain disease among adults by 2030 (1, 2). When individuals with CSDH develop evident clinical symptoms, conservative therapy is relatively ineffective and surgery is often the only option (3). At present, the main methods of operation for CSDH are craniotomy, twist drilling drainage, and burr hole surgery (4–6). Burr hole surgery is the most effective and simplest method of removing CSDH (7). Previous CSDH research has tended to focus on surgical approaches (7–10), but there have been few investigations on intra-operative anesthetic procedures. For CSDH burr hole surgery, there are two basic forms of anesthesia: local anesthesia (LA) (11) and general anesthesia (GA) (12). Elderly patients carry a higher perioperative risk and are associated with worse outcomes in CSDH (13), therefore, the choice of anesthesia mode is very important for elderly patients (11). However, there is no clear conclusion about how these two kinds of anesthesia impact the surgical procedure and recovery of patients; or which type of anesthesia is more suitable for CSDH burr hole surgery. The goal of this study was to explore the most suitable and effective anesthesia mode for the surgical operation and rehabilitation of patients with CSDH.

## Materials and Methods

This study was approved by the Institutional Review Board of Second Affiliated Hospital of Shantou University Medical College. For the retrospective nature of this study, obtaining informed patient consent was no more required.

### Patient Selection

Using the database of Second Affiliated Hospital of Shantou University Medical College, patients who have undergone single burr hole surgery for CSDH from 1^st^ January 2013 to 31^st^ December 2018 were identified; along with the type of anesthesia (local or general) used during these surgeries. Patients were retrospectively assigned to a LA or GA group according to the anesthesia method they chose. In the current study, patients chose their anesthesia mode by themselves after receiving the detailed explanations of the implementation process of the two anesthesia methods. Through preoperative evaluation, both local anesthesia and general anesthesia were confirmed to be applicable to all cases enrolled in this study. Patients who received local anesthesia, maintained with 1% lidocaine used for subcutaneous infiltration at the incision site at the beginning of the operation, were categorized as LA group. Patients who received general anesthesia, maintained with propofol-based total intravenous anesthesia with tracheal intubation during the surgery, were categorized as GA group. All of these patients had undergone burr hole surgery to remove the hematoma. All of the one burr hole surgeries included in this study were completed at the authors' institution. Both groups had the same surgical process, operation members, and materials utilized during the surgery. Before the procedure, all the patients gave their consent for this study.

The following inclusion criteria were considered: (1) unilateral CSDH as diagnosed by cranial computed tomography (CT), (2) obvious clinical symptoms, (3) required surgical treatment, and (4) the ability to cooperate with the surgeons during the surgery [Glasgow Coma Scale (GCS) 12–15]. The following exclusion criteria were considered: (1) bilateral CSDH, (2) cancer, (3) multiple organ dysfunction, (4) an inability to cooperate with the surgeons during the operation (GCS < 12 points), and (5) use of anticoagulants or antiplatelet agents.

### Mode of Anesthesia

#### The LA Group Was Treated With Local Anesthesia

One percent lidocaine was used for subcutaneous infiltration at the incision site at the beginning of the operation, and the patient received a full explanation of the procedure before the operation to obtain their understanding and cooperation during the operation.

#### The GA Group Received General Anesthesia Through Tracheal Intubation

Midazolam 2 mg, 1% propofol 7 ml, and fentanyl 0.1 mg were injected intravenously, and cisatracurium besylate 10 mg were injected intravenously after the patient fell asleep. While the patient stopped breathing with muscle relaxation, a tracheal tube was placed, and then the patient's breathing was continuously controlled by a ventilator. Moreover, 1% Propofol 1.5 ug/kg/min and Remifentanil 15 ug/kg/min were continuously maintained with a micropump until the end of the operation. Cisatracurium besylate was injected intravenously every 30 min during the operation. These drugs were discontinued at the end of the operation.

### Operation Procedure

The operation procedures were the same for both groups. The patient was lying on their back with their head resting on a horseshoe headrest. The patient's head was rotated by 45 degrees toward the healthy side and was given routine disinfection and towel lying as needed. On the thickest plane of the hematoma, a 3- to 4-cm scalp incision was done and the skull was pierced with a hole. Dark red blood fluid could be observed gushing from the hematoma cavity when the dura and the outer membrane of hematoma attached to the dura were opened. A small infusion tube, 2 mm in diameter, was inserted into the hematoma cavity (to better reach all parts of the hematoma cavity). The hematoma cavity was irrigated repeatedly through the small infusion tube in all directions with 0.9% saline until the fluids were clear. Finally, a drainage tube (Medtronic Inc, No. 14) was inserted gently into the hematoma cavity. The drainage tube reached the anterior of the hematoma to facilitate drainage of residual air in the hematoma cavity after the operation. The drainage tube was removed until the drainage volume was <10 ml/day (usually placed for 2–4 days). Patients in the LA group were told to cough when the hematoma was sticky and difficult to wash out during the operation, to promote the discharge of the hematoma.

### Data Recording

The anesthesia + operation time, the time from operation to discharge, hospitalization costs, postoperative complications, a retention time of the head drainage tube, residual hematomas, and hematoma recurrence (defined by a head CT reexamination showing an increase in hematoma volume compared with the first postoperative CT scan along with the patient having obvious clinical symptoms needing reoperation) were noted for the patients of both the groups and analyzed further statistically. The calculation of the hematoma volume was based on the Coniglobus formula, which has been shown in previous studies (14, 15), = 1/2 × the longest diameter of the hematoma layer with the largest area on axial sections (cm) × the longest diameter perpendicular to the longest diameter mentioned above (cm) × the hematoma thickness (cm).

### Postoperative Follow-Up

The duration of follow-up ranged from 1 month to 1 year, with an average of 6.1 months.

### Statistical Analysis

SPSS22.0 was used to analyze the data, and the *t*-test or chi-square test was used to compare the two groups. Quantitative data were expressed as mean ± standard deviation. Statistically, a *p* < 0.05 was considered a significant difference.

## Results

**Figure 1:**
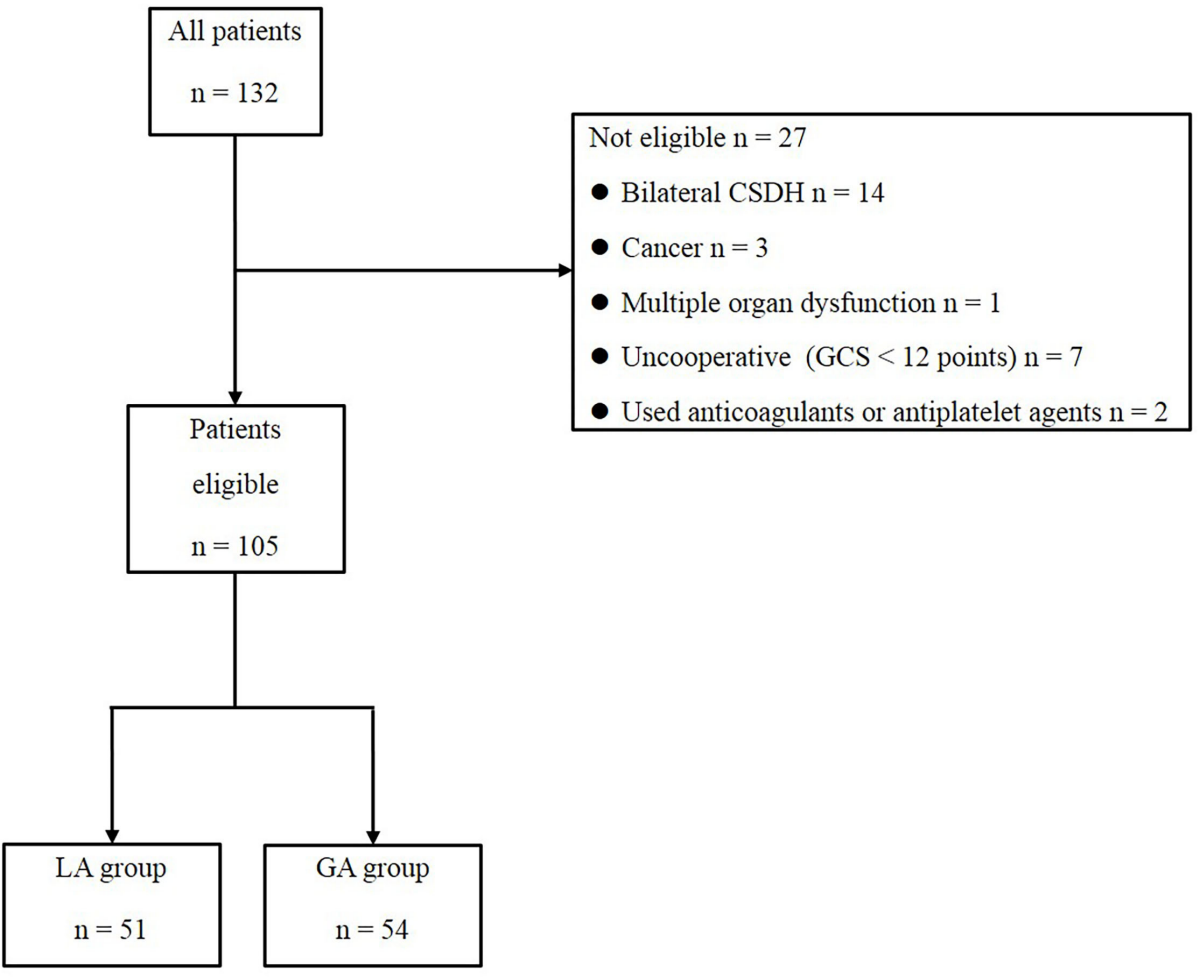
Flow chart for exclusion of data.

A total of 132 patients with CSDH, who underwent burr hole surgery between 1^st^ January 2013 and 31^st^ December 2018, were enrolled in this study (Figure 1). Following the exclusion of 27 individuals who did not meet the criteria, 105 individuals with unilateral CSDH were found to be eligible. Among them, there were 51 patients in the LA group and 54 patients in the GA group. The differences were not significant in age, sex, preoperative GCS score, CT value, side of hematoma, hematoma volume, and underlying diseases between the two groups before treatment (*P* > 0.05), as shown in Table 1. The preoperative symptoms of the patients in both groups are mentioned in Table 2. The most common symptoms of the patients included headache, dizziness, and weakness of limbs, slow reaction time, mental fatigue, vomiting, and slurred speech (Table 2). There were no significant differences in common symptoms of the patients between the two groups (*P* > 0.05).

**Table 1:** Baseline information.

**Variable**	**Entire cohort**	**LA group**	**GA group**	*P* value^*^
	**(*n* = 105)**	**(*n* = 51)**	**(*n* = 54)**	
Age, years, mean (SD)	64.50 (12.14)	62.25 (11.54)	66.63 (12.41)	0.065
Male sex, *n* (%)	91 (86.7)	46 (90.2)	45 (83.3)	0.393
GCS, score, mean (SD)	14.90 (0.39)	14.90 (0.36)	14.89 (0.42)	0.870
Left side, *n* (%)	60 (57.1)	32 (62.7)	28 (51.9)	0.325
Volume of hematoma, mL, mean (SD)	83.09 (16.26)	84.46 (16.90)	81.79 (15.67)	0.404
CT value, mean (SD)	33.95 (7.20)	32.60 (5.70)	35.21 (8.22)	0.063
Alcohol consumption, *n* (%)	23 (31.4)	12 (23.5)	11 (20.4)	0.814
Smoking, *n* (%)	31 (29.5)	17 (33.3)	14 (25.9)	0.521
Heart disease, *n* (%)	6 (5.7)	4 (7.8)	2 (3.7)	0.428
Hypertension, *n* (%)	17 (16.2)	7 (13.7)	10 (18.5)	0.600
Diabetes mellitus, *n* (%)	11 (10.5)	5 (9.8)	6 (11.1)	1.000
Pulmonary disease, *n* (%)	3 (2.9)	2 (3.9)	1 (1.9)	0.611
Cerebral lacunar infarction, *n* (%)	2 (1.9)	0 (0)	2 (3.7)	0.496
Chronic renal insufficiency, *n* (%)	1 (1.0)	1 (2.0)	0 (0)	0.486
Gout, *n* (%)	1 (1.0)	1 (2.0)	0 (0)	0.486
Forearm fracture, *n* (%)	1 (1.0)	1 (2.0)	0 (0)	0.486
Rheumatoid arthritis, *n* (%)	1 (1.0)	0 (0)	1 (1.9)	1.000
Epilepsy, *n* (%)	1 (1.0)	0 (0)	1 (1.9)	1.000
Anemia, *n* (%)	1 (1.0)	0 (0)	1 (1.9)	1.000

* *P value represents comparison between the two groups. Means are compared with T-tests, proportions with the Chi-square test. LA, local anesthesia; GA, general anesthesia*.

**Table 2:** Preoperative symptoms.

**Variable**	**Entire cohort**	**LA group**	**GA group**	*P* value^*^
	**(*n* = 105)**	**(*n* = 51)**	**(*n* = 54)**	
Weakness of limbs, *n* (%)	77 (73.3)	37 (72.5)	40 (74.1)	1.000
Slow reaction, *n* (%)	9 (8.6)	5 (9.8)	4 (7.4)	0.737
Dizziness, *n* (%)	54 (51.4)	28 (54.9)	26 (48.1)	0.560
Headache, *n* (%)	37 (35.2)	20 (39.2)	17 (31.5)	0.422
Vomiting, *n* (%)	5 (4.8)	3 (5.9)	2 (3.7)	0.672
Paraphasia, *n* (%)	1 (1.0)	1 (2.0)	0 (0)	0.486
Slurred speech, *n* (%)	5 (4.8)	2 (3.9)	3 (5.6)	1.000
Declines in memory, *n* (%)	1 (1.0)	0 (0)	1 (1.9)	1.000
Involuntary movement, *n* (%)	2 (1.9)	0 (0)	2 (3.7)	0.496

* *P value represents comparison between the two groups. Proportions are compared with the Chi-square test*.

In both groups, the operations were completed smoothly and successfully. No initial LA patients had to be switched to GA due to their uncooperative behavior. The anesthesia and operation time of the LA patients was 37.71 (10.55) min, and that of GA patients was 56.04 (8.37) min; the difference being statistically significant (*p* < 0.001). The time duration from operation to discharge in the LA group was 8.51 (1.49) days, which was significantly shorter than that in GA patients [10.46 (2.34) days; *p* < 0.001]. The total cost of hospitalization for LA patients was 2,721.54 (504.66) USD, which was significantly less than that for GA patients [3,314.82 (493.52) USD; *p* < 0.001]. The retention time of the head drainage tube in the LA group was shorter than that in the GA group [(2.55 (0.88) days vs. 3.04 (0.89) days, respectively; *p* = 0.006]. The number of patients with residual hematoma in the LA group was less than that in the GA group (*p* = 0.014). However, significant difference between the two groups was not found between the two groups in recurrence rate (*p* = 1.000) (Table 3).

**Table 3:** Postoperative information.

**Variable**	**Entire cohort**	**LA group**	**GA group**	*P* value^*^
	**(*n* = 105)**	**(*n* = 51)**	**(*n* = 54)**	
Anesthesia + Operation Time, minute, mean (SD)	47.13 (13.19)	37.71 (10.55)	56.04 (8.37)	<0.001
Duration from operation to discharge, day, mean (SD)	9.51 (2.19)	8.51 (1.49)	10.46 (2.34)	<0.001
Hospitalization cost, USD, mean (SD)	3,026.66 (579.08)	2,721.54 (504.66)	3,314.82 (493.52)	<0.001
Retention time of head drainage tube, day, mean (SD)	2.80 (0.91)	2.55 (0.88)	3.04 (0.89)	0.006
Volume of residual hematoma (first day after operation):				0.012
None, *n* (%)	9 (8.6)	8 (15.7)	1 (1.9)	
Little <10 mL, *n* (%)	71 (67.6)	36 (70.6)	35 (64.8)	
Middle 10–20 mL, *n* (%)	23 (21.9)	7 (13.7)	16 (29.6)	
Large>20 mL, *n* (%)	2 (1.9)	0 (0)	2 (3.7)	
Total cases of residual hematoma, *n* (%)	96 (91.4)	43 (84.3)	53 (98.1)	0.014
Operative recurrence, *n* (%)	9 (8.6)	4 (7.8)	5 (9.3)	1.000

* *P value represents comparison between the two groups. Means are compared with T-tests, proportions with the Chi-square test*.

The main complications observed in the GA group were nausea and vomiting, dyspnea, pneumonia, delayed awakening, and restlessness. While the major complications in the LA group patients were nausea and vomiting, poor wound healing, and brain tissue injury by drainage tube insertion. There were only six cases of complications in LA patients, which has a significantly smaller amount than that of GA patients (*p* < 0.001) (Table 4). After surgery, all of the patients were released with improved symptoms, and no deaths were reported in any of the groups.

**Table 4:** Postoperative complications.

**Variable**	**Entire cohort**	**LA group**	**GA group**	*P* value^*^
	**(*n* = 105)**	**(*n* = 51)**	**(*n* = 54)**	
Pneumonia, *n* (%)	3 (2.9)	0 (0)	3 (5.6)	0.243
Restlessness, *n* (%)	4 (3.8)	0 (0)	4 (7.4)	0.118
Vomiting, *n* (%)	9 (8.6)	2 (3.9)	7 (13.0)	0.162
Sore throat, *n* (%)	2 (1.9)	0 (0)	2 (3.7)	0.496
Delayed Awakening, *n* (%)	4 (3.8)	0 (0)	4 (7.4)	0.118
Epilepsy, *n* (%)	2 (1.9)	1 (2.0)	1 (1.9)	1.000
Poor wound healing, *n* (%)	1 (1.0)	1 (2.0)	0 (0)	0.486
Dyspnea, *n* (%)	6 (5.7)	0 (0)	6 (11.1)	0.027
Drainage tube insertion into brain tissue, *n* (%)	3 (2.9)	2 (3.9)	1 (1.9)	0.611
Intracranial infection, *n* (%)	1 (1.0)	0 (0)	1 (1.9)	1.000
Total complications	35	6	29	<0.001

* *P value represents comparison between the two groups. Proportions are compared with the Chi-square test*.

## Discussion

From this current study, it was found that burr hole surgery performed under assisted local anesthesia is safe and more beneficial for patients with CSDH, which was consistent with Seizeur's research (11). Duration of operation (including anesthesia and operating time) in the LA group was much shorter than that in the GA group. The explanation for it is that anesthesia in the LA group does not require continuous intravenous administration of anesthetics to keep the patient sedated, analgesic, and unconscious. As a result, there is no need for anesthetic induction and recovery, as well as tracheal intubation and extubation. Due to residual anesthetics and hemodynamic changes produced by anesthetics, postoperative problems in the GA group were substantially greater than those in the LA group. This study found that patients in the GA group had more respiratory depression and pulmonary infection than those in the LA group. Six patients in the GA group experienced dyspnea after surgery, two of which were due to secretion reflux inhalation into the respiratory tract (the secretion was difficult for patients to cough out); three were due to glossoptosis with the tongue obstructing the throat; and one was due to difficulty in tracheal intubation, where repeated stimulation resulted in laryngeal spasms and also led to a postoperative sore throat. Although the complications of GA, such as airway obstruction and respiratory depression, are mostly reversible, and not all patients will progress to pulmonary infection; the occurrence of similar complications will increase the risk of patients' prolonged hospitalization time suffering and increase their hospitalization costs.

Previous studies revealed that some patients have delayed awakening, hallucinations, mania, and other restless phenomena when GA was administered due to the residual anesthetics (16, 17). In this study, four patients in the GA group had delayed recovery and restlessness after surgery; nausea and vomiting were also common amongst this group. The most common cause of postoperative vomiting is anesthesia side effects and aspiration of vomit is typical, resulting in respiratory obstruction. Blood pressure fluctuations before and after surgery, as well as arrhythmia, are typical problems in GA patients (18). It has also been reported that a high-dose combination of propofol and remifentanil during anesthesia can decrease blood pressure and heart rate, and cause hypoxia and respiratory arrest, which increase the risk of death (19). During this study it was observed that patients in the LA group had no difficulty in breathing, no restlessness, no delayed awakening after the operation; and only a few patients had nausea and vomiting as compared to the GA group. LA patients were also not at risk for airway injury and pain because they do not need to receive tracheal intubation. As LA treatment does not include muscle relaxants or sedatives, these individuals are not at risk for respiratory depression or deleterious effects on blood circulation or gastrointestinal function. In addition, the earlier post-surgery independence of eating and movement also guaranteed better recovery for the patients.

The hospitalization time of the LA patients was significantly shorter than that of the GA patients, which has ultimately reduced their hospitalization costs and the risk of hospital infection. So, LA is particularly suitable for elderly and infirm patients with multiple disorders as well as patients who do not wish to undergo surgery using GA. In contrast to patients with GA, patients with LA can cough to promote hematoma discharge if the hematoma discharge is unsatisfactory during the surgery, which explains why the LA group has fewer patients with residual hematoma after the surgery.

To date, only a few reports are investigating CSDH removal under local anesthesia (20–22). A recent study showed that a series of patients with unilateral and bilateral CSDH successfully underwent burr hole surgery under LA in the sitting position, indicating that LA is safe for the patient with CSDH, even for bilateral CSDH (22). It was found that midazolam sedation combined with LA can achieve the same anesthetic effect as GA during mini-craniotomy of CSDH (21). However, the sedatives employed in the aforementioned method might cause respiratory and cardiac side effects, as well as restlessness and unwillingness to co-operate in some patients during the operation (20). An increased dose of sedatives to calm down such patients may result in deep sedation, respiratory depression, and other complications. Ultimately, some patients may be required to switch to GA with tracheal intubation. The second study showed that three of the patients could not cooperate with the operation under LA combined with sedation, and one of them had to be switched to GA with tracheal intubation; whereas, the other two patients needed a laryngeal mask to be inserted to relieve dyspnea. The failure rate of dexmedetomidine combined with LA for CSDH burr hole surgery was found to be 7.9% (12). Such change from LA to GA with intubation during the surgery makes the whole operation procedure very passive. It delays the operative time and might also cause intracranial or incision infection combined with other complications, causing potential risks to patients.

Most of the patients in this research showed residual hematoma on a CT scan after the operation. However, CT reexamination revealed that most of the residual hematomas were gradually absorbed, with just a few patients requiring reoperation. Previous studies have found that placing a drainage tube under the subgaleal space can play a positive role in draining a hematoma (23), indicating that residual hematomas may flow out of the dural break and gradually infiltrate into the subgaleal space for absorption when brain pulsation and body position changes. Hence, enlarging the incision of the dura mater and hematoma capsule may promote hematoma discharge. Furthermore, the direction of the small infusion tube used during the operation can be changed more flexibly in the hematoma cavity and reach the depth of the hematoma cavity easily, making it more effective to wash out the hematoma than to wash the hematoma cavity directly with a large external drainage tube. During this study, three cases of brain tissue injury caused by the insertion of the drainage tube were reported, as the hematoma cavity was small, making it difficult to insert the tube. To avoid such injury, saline solution was injected to expand the hematoma cavity to aid in drainage tube insertion. A recent randomized clinical study has shown that subperiosteal drain (SPD) is considered to be safer as drain misplacement rates were lower, owing to SPD is not positioned in direct contact to cortical structures (9).

There are still some limitations to this study. Firstly, the patient number in the current study who underwent treatment was too small, which weakened the conclusion of this study that local anesthesia is a safer and more effective anesthesia method than general anesthesia for burr hole surgery of CSDH. Secondly, the major limitation of this study is the retrospective nature, which might lead to a risk of bias. Since this study is a preliminary study from a single center, further multi-center randomized clinical trials should be established with a better follow-up system in order to get more precise conclusions.

## Conclusion

From this retrospective study, it was found that CSDH burr hole surgery using local anesthesia might shorten anesthesia and operation time, and hospitalization time, less postoperative complications; ultimately reducing hospitalization costs of the patients as compared to surgery using general anesthesia. It seems that CSDH burr hole surgery using local anesthesia might be beneficial for unilateral CSDH patients with a GCS >12. Further research is still required to confirm this conclusion.

## Data Availability Statement

The raw data supporting the conclusions of this article will be made available by the authors, without undue reservation.

## Ethics Statement

The studies involving human participants were reviewed and the present study was approved by the Institutional Review Board of Second Affiliated Hospital of Shantou University Medical College. Written informed consent for participation was not required for this study in accordance with the national legislation and the institutional requirements.

## Author Contributions

JL and AL: conception and design. ZC and JZ: acquisition of data. ZC, HC, BC, and JZ: analysis and interpretation of data. JL and ZZ: drafting the article and critically revising the article. All authors contributed to the article and approved the submitted version.

## Funding

This study was supported by the Natural Science Foundation of Guangdong Province of China [Grant No. 2021A1515011179] and Science and Technology Planning Project of Guangdong Province of China [Grant No. 2021(88)-4].

## Conflict of Interest

The authors declare that the research was conducted in the absence of any commercial or financial relationships that could be construed as a potential conflict of interest.

## Publisher's Note

All claims expressed in this article are solely those of the authors and do not necessarily represent those of their affiliated organizations, or those of the publisher, the editors and the reviewers. Any product that may be evaluated in this article, or claim that may be made by its manufacturer, is not guaranteed or endorsed by the publisher.
